# An hourglass mechanism controls torpor bout length in hibernating garden dormice

**DOI:** 10.1242/jeb.243456

**Published:** 2021-12-09

**Authors:** Thomas Ruf, Kristina Gasch, Gabrielle Stalder, Hanno Gerritsmann, Sylvain Giroud

**Affiliations:** Research Institute of Wildlife Ecology, Department of Interdisciplinary Life Sciences, University of Veterinary Medicine, Savoyenstrasse 1, A-1160 Vienna, Austria

**Keywords:** Cycles, Interbout euthermia, Metabolic rate, Periodic arousal

## Abstract

Hibernating mammals drastically lower their rate of oxygen consumption and body temperature (*T*_b_) for several weeks, but regularly rewarm and stay euthermic for brief periods (<30 h). It has been hypothesized that these periodic arousals are driven by the development of a metabolic imbalance during torpor; that is, the accumulation or the depletion of metabolites or the accrual of cellular damage that can be eliminated only in the euthermic state. We obtained oxygen consumption (as a proxy of metabolic rate) and *T*_b_ at 7 min intervals over entire torpor–arousal cycles in the garden dormouse (*Eliomys quercinus*). Torpor bout duration was highly dependent on mean oxygen consumption during the torpor bout. Oxygen consumption during torpor, in turn, was elevated by *T*_b_, which fluctuated only slightly in dormice kept at ∼3–8°C. This corresponds to a well-known effect of higher *T*_b_ on shortening torpor bout lengths in hibernators. Arousal duration was independent from prior torpor length, but arousal mean oxygen consumption increased with prior torpor *T*_b_. These results, particularly the effect of torpor oxygen consumption on torpor bout length, point to an hourglass mechanism of torpor control, i.e. the correction of a metabolic imbalance during arousal. This conclusion is in line with previous comparative studies providing evidence for significant interspecific inverse relationships between the duration of torpor bouts and metabolism in torpor. Thus, a simple hourglass mechanism is sufficient to explain torpor/arousal cycles, without the need to involve non-temperature-compensated circadian rhythms.

## Abstract (precis)

**Summary:** In hibernating garden dormice, torpor bout length depends on oxygen consumption. This indicates that torpor duration is determined by accumulation of a metabolic imbalance, which is cleared during periodic rewarming.

## INTRODUCTION

**Fig. 1.**
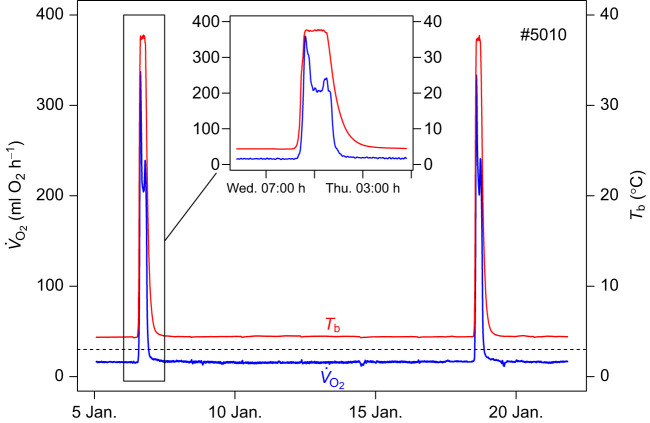
**Example of oxygen consumption during torpor and two arousals (with interbout euthermia) in a garden dormouse (animal #5010).** The duration of torpid phases and of arousals was determined from metabolic rate (measured as oxygen consumption rate, *V̇*_O_2__) crossing a threshold of 30 ml O_2_ h^−1^ (dashed line; set by visual inspection). Mean metabolic rate was computed from all data points falling above (arousal) or below (torpor) this threshold. The inset shows the first arousal on an amplified time scale. Arousals were characterized by an initial burst of oxygen consumption. In this case, there was a second burst of oxygen consumption prior to torpor entrance (during the second half of the arousal), with a height termed late peak *V̇*_O_2__. The red lines show *T*_b_ of the animal.

Hibernation in mammals is characterized by a profound reduction of metabolic rate, often to a level of ≤5% of basal metabolic rate (BMR) (Ruf and Geiser, 2015). Typically, this decrease of metabolic rate is accompanied by a reduction of body temperature (*T*_b_) to values just above ambient temperature (*T*_a_). However, most hibernators do not maintain low metabolic rate and *T*_b_ throughout winter. Apart from a few species that can continually hibernate at a *T*_b_ of approximately 30°C or even above that (Dausmann et al., 2004; Tøien et al., 2011), hibernating mammals regularly rewarm from the torpid to the euthermic state during so-called spontaneous arousals (Fig. 1). The maximum duration of torpor bouts is a species-specific trait and varies from ∼3 to 98 days (Ruf and Geiser, 2015). Rewarming and subsequent intervals of interbout euthermia are responsible for at least 70% of the total energy expenditure over winter (Wang, 1979). However, since the first discovery of these spontaneous ‘periodic changes’ of *T*_b_ (Hall, 1832), their function has remained unclear. Among other hypotheses, it has been suggested that hibernators rewarm in order to sleep (Daan et al., 1991; Trachsel et al., 1991), to combat pathogens (Prendergast et al., 2002) or to restore enzymes required for cardiac function at low *T*_b_ (Ruf and Arnold, 2008). However, the ‘warming up for sleep’ hypothesis has been refuted (Larkin and Heller, 1998; Strijkstra and Daan, 1998), and the other hypotheses remain speculative. Similarly, the clock mechanisms that control the timing of torpor and arousal within the hibernation season are entirely unknown, and even their fundamental nature is a question of debate (Malan, 2010; Ruf and Geiser, 2015).

Over several decades, it had been assumed repeatedly that the torpor–arousal cycle is driven by a so-called hourglass mechanism. This hypothesis assumes the development of a metabolic imbalance during torpor; that is, the accumulation or depletion of metabolites or the accrual of cellular damage that can be eliminated only in the euthermic state (Carey et al., 2003; Fisher, 1964; French, 1985; Galster and Morrison, 1976; Lyman et al., 1982; Martin and Epperson, 2008; Strijkstra, 1999; Twente and Twente, 1968; Van Breukelen and Martin, 2002b). It seems, however, that at the low *T*_b_ of deep torpor, the continued degradation and depletion of metabolites is much more likely than their energy-consuming production and accumulation. An effect of metabolism on torpor bout length also seemed to be supported by a study by French (1985) that pointed to a decrease of torpor bout duration with body mass – and, by inference, oxygen consumption increase – albeit in a limited sample of mammals. A more recent comparative study showed that torpor bout duration among mammals in fact decreases as minimum oxygen consumption in torpor increases (Ruf and Geiser, 2015). This result is fully compatible with the idea of a metabolic imbalance, such as a metabolite deficiency, that is formed faster if torpor oxygen consumption is high. Also, the hourglass hypothesis seemed to be supported, for instance, by the observation that in ground squirrels an increase in oxygen consumption during torpor, due to animals defending a setpoint *T*_b_ at very low *T*_a_, is associated with a shortening of torpor bout duration (Buck and Barnes, 2000; Geiser and Kenagy, 1988). However, the conclusiveness of these studies was somewhat limited by the fact that measurements were either restricted to certain time points during torpor episodes (Geiser and Kenagy, 1988), or that *T*_b_ and oxygen consumption were obtained from different individuals from those for torpor bout duration (Buck and Barnes, 2000).

The main reason why the existence of such an hourglass mechanism was dismissed in the past was the complete absence of an effect of body mass on torpor duration, when hibernating animals were compared (Geiser and Ruf, 1995; Malan, 2010; Ruf and Geiser, 2015). Malan (2010) argued that the absence of an effect of body mass on torpor bout duration is reason to refute the hourglass hypothesis. Indeed, as metabolic rate is usually strongly affected by body mass, it seems logical to assume that independence of torpor bout duration from body mass also should reflect independence of the torpor–arousal cycle from metabolism. Instead of an hourglass mechanism, Malan (2010) therefore proposed the existence of a specialized, non-temperature-compensated circadian clock that governs torpor–arousal cycles.

Therefore, here we obtained data on entire torpor–arousal cycles in the garden dormouse (*Eliomys quercinus*), a medium-sized hibernator (∼100 g). We hypothesized that, if an hourglass is the governing mechanism, torpor bout duration should decrease with increasing mean torpor oxygen consumption measured over the entire torpor bout in the same individual. We also aimed to test whether torpor bout duration is affected by previous arousals, e.g. by the duration or oxygen consumption during arousals. We further hypothesized that the duration or oxygen consumption during arousals may be affected by the previous torpor bout, if an hourglass mechanism is at work. Alternatively, if the torpor–arousal cycle is governed by a non-temperature-compensated circadian clock, the duration of torpor bouts should not be affected by oxygen consumption.

## MATERIALS AND METHODS

### Animals and housing

The garden dormouse, *Eliomys quercinus* (Linnaeus 1766), is a nocturnal, arboreal and omnivorous rodent widely distributed in Europe. Garden dormice show deep hibernation with oxygen consumption depression down to <2% compared with their euthermic state while *T*_b_ reaches 1°C. The maximum duration of torpor bouts in this species is 20 days; the average during midwinter is 14 days (Ruf and Geiser, 2015).

The adult garden dormice used in this study were bred and raised at the Research Institute of Wildlife Ecology (FIWI), Vienna, Austria (latitude 48°15′N, longitude 16°22′E). Animals were reared in outdoor enclosures under natural variations of photoperiod and *T*_a_. Prior to hibernation, dormice were housed separately in polycarbonate cages (60×40×40 cm) and had access to food (Altromin 7024, Altromin GmbH & Co. KG, Lage, Germany) and water *ad libitum*. The dormice were also fed with sunflower seeds and dry insects twice a week. During the experiments, the animals had no access to food to simulate a natural situation of winter hibernation. The experiments involved 22 garden dormice with body mass ranging from 75 to 169 g prior to hibernation.

### Experiments

Experiments were carried out between November 2014 and April 2016. The rate of oxygen consumption (*V̇*_O_2__) was measured in garden dormice during torpor by indirect calorimetry. Dormice were kept individually in a ventilated Perspex respiratory chamber (volume 5.4 l) supplied with fresh air. Respirometry chambers were placed inside refrigerators set to +5°C, but fluctuated with the refrigerator control (range 2.9–7.8°C, s.d. 0.67°C). The temperature inside the refrigerators was measured with small (∼2 g) temperature loggers (custom made and calibrated at the Research Institute of Wildlife Ecology; accuracy ±0.1°C). Core *T*_b_ of each animal was continuously recorded with transmitters (model: TA-10TA-F20, 1.75 cc, 3.8 g, accuracy: 0.15°C; Data Sciences International, Saint Paul, MN, USA). Transmitters were calibrated prior to implantation between 0 and 40°C in a temperature-controlled water bath. The transmitters were surgically implanted under anaesthesia as described in detail elsewhere (Giroud et al., 2018). A receiver board (RPC-1; Data Sciences International) was positioned under each individual cage to collect the radio frequency signals from transmitters. *T*_b_ was recorded for 10 s every 5 min. Each animal was held in the respiratory chamber until at least two arousal phases were recorded. The animals measured were removed from the refrigerators as part of another experiment (Huber et al., 2021). Therefore, we obtained a varying number of measurements from each animal. We obtained 114 records of interbout euthermia together with the complete preceding or subsequent torpor bout from 22 dormice. All animals were weighed before and after the experiment (but before re-feeding) to the nearest 0.1 g (CS 200, Ohaus, Parsippany, NJ, USA).

### Metabolic rate measurements

Metabolic rate was measured as *V̇*_O_2__ determined by a dual-channel oxygen analyser (Moxzilla, Sable Systems, Las Vegas, NV, USA). The analyser was calibrated using a high-precision gas-proportioning pump (type 55A27/7a, H. Wösthoff, Bochum, Germany). Flow rates through airtight respirometry chambers were measured with calibrated mass flow meters (FMA 3100, Omega Engineering, Stamford, CT, USA). A gas multiplexer enabled airflow to be switched between 6 animal chambers at 1 min intervals. A 7th empty respirometry chamber supplied with fresh air was recorded to continually correct for drift. Thus, a *V̇*_O_2__ reading was recorded per animal at 7 min intervals. Air leaving the respirometry chambers was not dried but relative humidity was measured (RH300, Sable Systems) and corrected for. All recordings were interfaced to a computer (Labjack U6, Lakewood, CO, USA) and *V̇*_O_2__ was calculated by a custom-written Python program based on equations given in Lighton (2008).

### Data analysis

To determine torpor bout and arousal length, we used a threshold of 30 ml O_2_ h^−1^, slightly above torpor *V̇*_O_2__ in all animals (chosen by visual inspection; Fig. 1). From the times when *V̇*_O_2__ crossed this threshold, we calculated arousal duration as well as torpor bout duration. We also computed mean *V̇*_O_2__, *T*_b_ and *T*_a_ for these phases. Consequently, these variables include a small number of points during transitions, i.e. rewarming from and entrance into torpor (Fig. 1). We included these points because oxygen consumption during these transitions may well affect a putative hourglass mechanism. We also recorded hibernation duration as the time between first placing the animals in cold chambers and the onset of each torpor bout.

Because of rapid rewarming, arousals always started with a burst of oxygen consumption (Fig. 1, inset). In some cases, the animals also displayed a second burst of oxygen consumption towards the end of arousals prior to re-entrance into torpor. We measured the amplitude of the peaks in oxygen consumption rate (late peak *V̇*_O_2__) by averaging the three highest values during the second half of an arousal. Dormice deplete body fat reserves during hibernation. Therefore, the body masses used in statistical analyses were computed by linearly interpolating body mass between the onset of the hibernation season and termination of measurements at the time of each arousal. We also computed means of *T*_b_ during torpor (in previous and subsequent bouts relative to the arousal, *T*_b,pre_ and *T*_b,sub_) as well as *T*_a_ during prior torpor bouts (*T*_a,pre_) and during arousal (arousal *T*_a_). Because of collinearity (variance inflation factor, VIF ∼8), we entered only one *T*_a_ measurement in the statistical analysis.

**Fig. 2.**
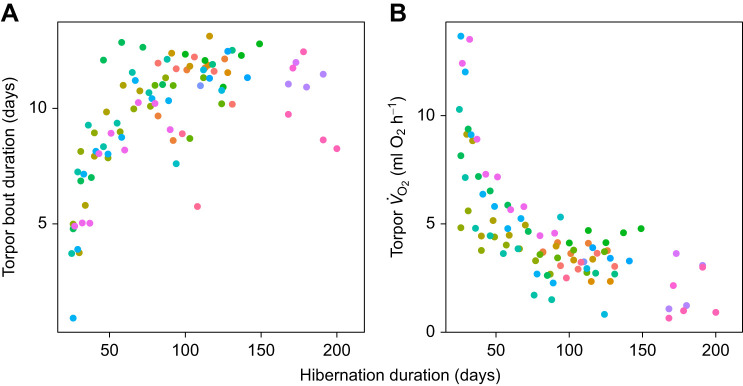
**Torpor bout duration and mean *V̇*_O_2__ in torpor as a function of time since the onset of hibernation in garden dormice.** While torpor duration (A) gradually increased, torpor *V̇*_O_2__ (B) decreased. Circles of the same colour are data from the same animals. Data from 94 torpor–arousal cycles in 22 animals.

The data comprised multiple individual torpor–arousal cycles from the same animal, ranging from 1 to 9 torpor–arousal cycles per animal (Fig. 2). Therefore, we used generalized linear mixed models (GLMMs), with separate intercepts for individuals to adjust for repeated measurements. GLMMs were computed using R 4.0.2 (http://www.R-project.org/), specifically the packages ‘brms’ (Bürkner, 2017, 2018) and ‘rstan’ (https://cran.r-project.org/web/packages/rstan/vignettes/rstan.html). The Bayesian GLMM approach implemented in these libraries has the advantage that it can readily estimate random effects even when data are only partly obtained as repeated measures. This data structure often causes singularities and prevents random effect estimates with other methods. Also, Bayesian analysis provides inferences that are conditional on the data and are exact, without reliance on asymptotic approximation (SAS Institute Inc., 2018). We provide posterior parameter distributions, their mean as well as 95% credible intervals (CI), and a Bayesian version of *R*^2^ for regression models (Table 1).

**Table 1.**
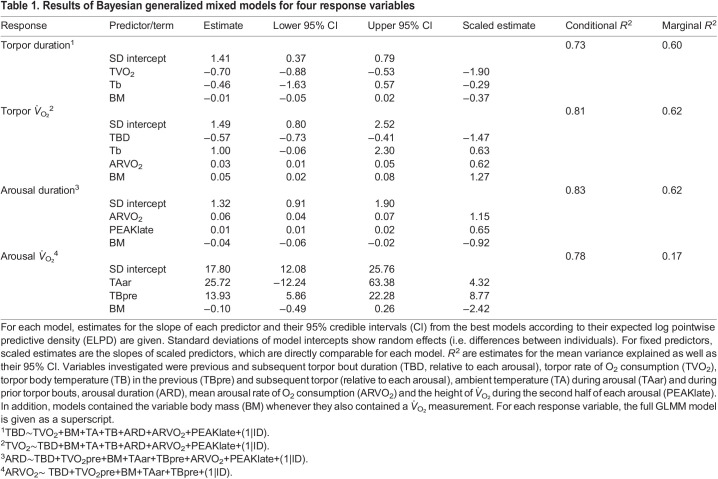
Results of Bayesian generalized mixed models for four response variables

All GLMM samples were drawn with the No-U-Turn-Sampling (NUTS) algorithm using 4 chains and 4000 iterations (including 2000 iterations per chain for warmup). We visually inspected MCMC chain plots and only report models for which the convergence diagnostic, Rhat, was 0.95–1.05. The response variables and corresponding full models are given in Table 1. Because the evaluation of all possible predictors was computationally not feasible, we reduced models in a stepwise procedure. Terms were eliminated to determine the model that maximized the expected log pointwise predictive density (ELPD) using the function ‘loo_compare’, which is based on leave-one-out cross-validation (https://rdrr.io/cran/loo/). However, in all models containing *V̇*_O_2__, we kept body mass as a fixed predictor in order to adjust metabolic rates for mass effects while avoiding the use of indices (Fernández-Verdejo et al., 2019). We used only weakly informative priors (the default priors in brms), as we had no prior information on expected slopes, and to avoid bias on the resulting posterior distributions (Gelman et al., 2014; Kruschke, 2015).

To assess the relative importance of explanatory variables (within each response variable), models were recomputed with scaled variables (i.e. after subtracting the mean and dividing by the standard deviation). We did not assess interactions between predictor variables, as this would have resulted in severe overfitting of the limited dataset. All response variables were approximately normally distributed as confirmed by quantile–quantile plots, and we used family ‘gaussian’ for brms fits.

Data are available from Phaidra (https://phaidra.vetmeduni.ac.at/o:898).

### Ethics statement

All procedures were approved by the institutional ethics committee and the national Austrian authority according to §26 of Law for Animal Experiments, Tierversuchsgesetz 2012 – TGV 2012 (BMBWF-68.205/0137-WF/V/3b/2014).

## RESULTS

### Torpor bout duration

**Fig. 3.**
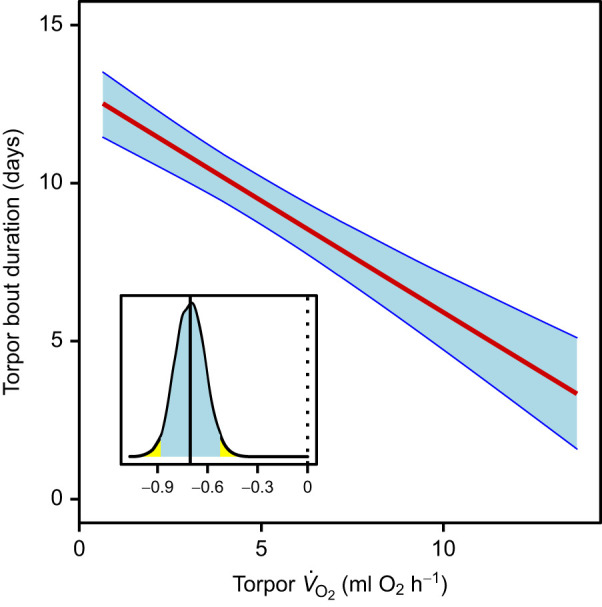
**Torpor bout duration decreased as mean torpor *V̇*_O_2__ increased.** Results of a Bayesian GLMM. Blue shaded areas indicate the 95% credible intervals of the predicted values at each value of torpor *V̇*_O_2__. The inset shows the posterior distribution of the slope estimate, its mean (solid line) and its 95% credible interval (shaded); the dashed line indicates a slope of zero. Data from 94 torpor–arousal cycles in 22 animals.

Torpor bout length increased progressively and torpor *V̇*_O_2__ declined over the hibernation season (Fig. 2). A regression of these variables showed a strong decrease of torpor bout duration as torpor bout *V̇*_O_2__ increased, and torpor bout *V̇*_O_2__ was the dominating variable determining the duration of torpor episodes (Table 1, Figs 2 and 3). Torpor *T*_b_ also remained in the best model of torpor bout duration, but the 95% CI of the *T*_b_ effect overlapped zero. Body mass was kept as a fixed factor, but its 95% CI also included zero (Table 1). Mean torpor bout duration was 10.34+3.69 days. The variation in torpor bout duration intercepts among individuals was moderate: the marginal *R*^2^ (fixed effects only) of the best model was 0.60, the conditional *R*^2^ (including the random part) was 0.73.

### Torpor *V̇*_O_2__

**Fig. 4.**
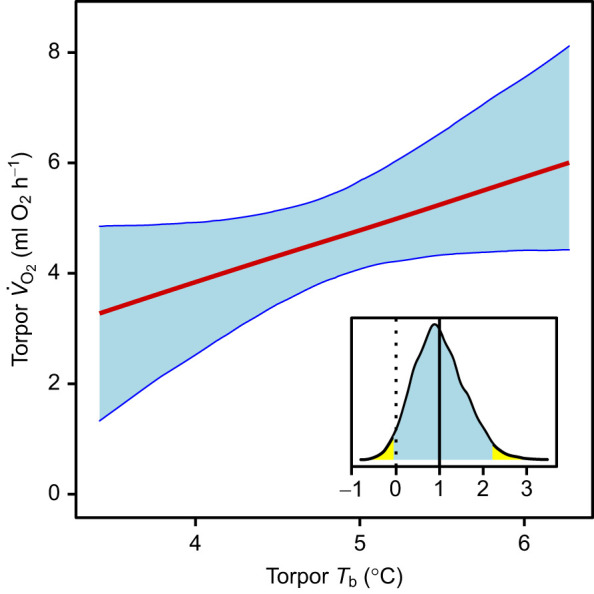
**Torpor *V̇*_O_2__ as a function of *T*_b_.** Results of a Bayesian GLMM. Blue shaded areas indicate the 95% credible intervals of the predicted values. The inset shows the posterior distribution of the slope estimate, its mean (solid lines) and 95% credible interval (shaded); the dashed line indicates a slope of zero. Data from 94 torpor–arousal cycles in 22 animals.

As expected from the above relationship, torpor *V̇*_O_2__ was negatively related to torpor bout duration (Table 1). Torpor *V̇*_O_2__ also tended to rise with mean torpor *T*_b_, with the 95% CI for the slope just overlapping zero (Fig. 4). Torpor *V̇*_O_2__ was also associated with average *V̇*_O_2__ during the previous arousal, which showed large differences between individuals (see below; Table 1). As expected, higher body mass also elevated total torpor *V̇*_O_2__ (Table 1). As indicated by the *R*^2^ (Table 1), approximately 20% of the variance was due to individual differences in torpor bout *V̇*_O_2__.

### Arousal duration

**Fig. 5.**
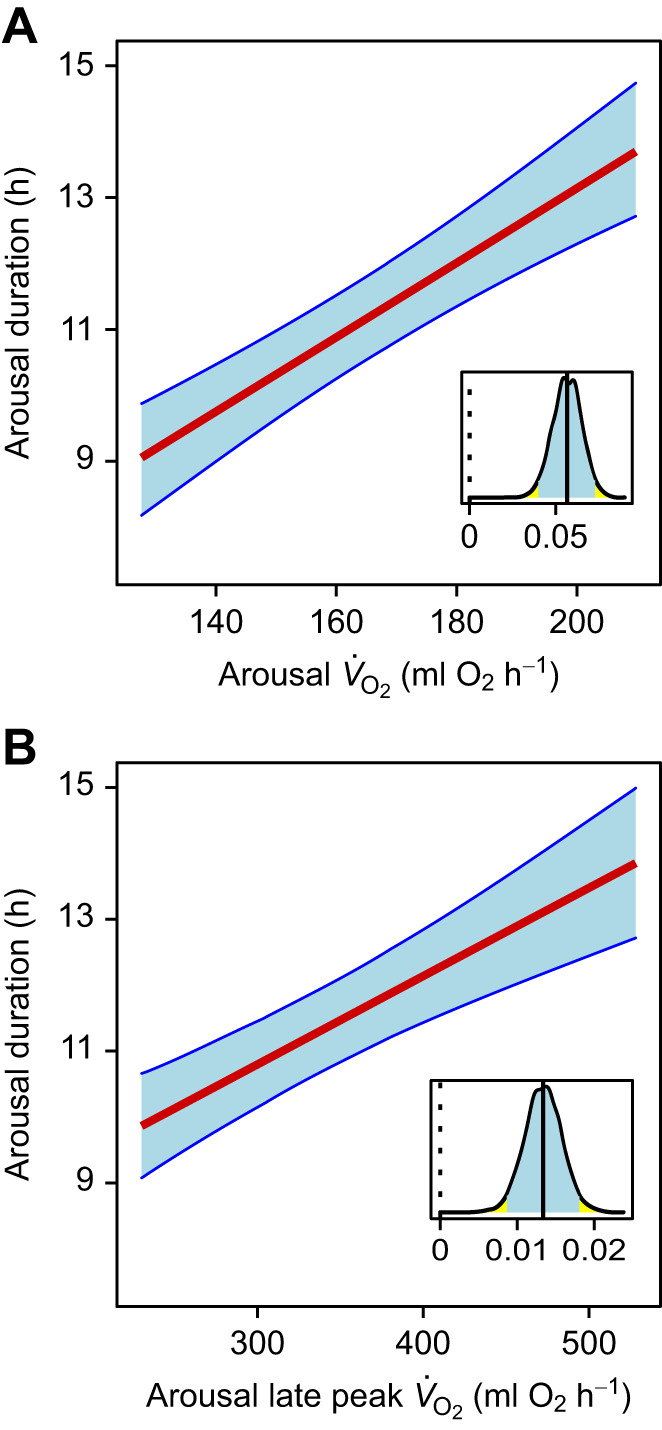
**Arousal duration as a function of mean *V̇*_O_2__ during the same arousal and the height of the metabolic burst in the second half of the arousal.** Results of a Bayesian GLMM for (A) arousal *V̇*_O_2__ and arousal late peak *V̇*_O_2__ (B). Blue shaded areas indicate the 95% credible intervals of the predicted values. The inset shows the posterior distribution of the slope estimate, its mean (solid line) and 95% credible interval (shaded). Data from 94 torpor–arousal cycles in 22 animals.

Arousals, including the re-warming and re-entrance phases, lasted from 8.2 to 16.7 h and were longer if *V̇*_O_2__ during the arousal episode was high (Fig. 5A). Higher bursts of oxygen consumption during the last half of the arousal (late peak *V̇*_O_2__) also prolonged its duration (Fig. 5B), while increased body mass had a shortening effect (Table 1). The mean duration of arousals was 11.10±0.15 h, and was completely independent from the prior torpor bout. Including the random factor individual had a moderate effect, as the marginal *R*^2^ of the best model was 0.62, whereas the conditional *R*^2^ was 0.83.

### Arousal *V̇*_O_2__

**Fig. 6.**
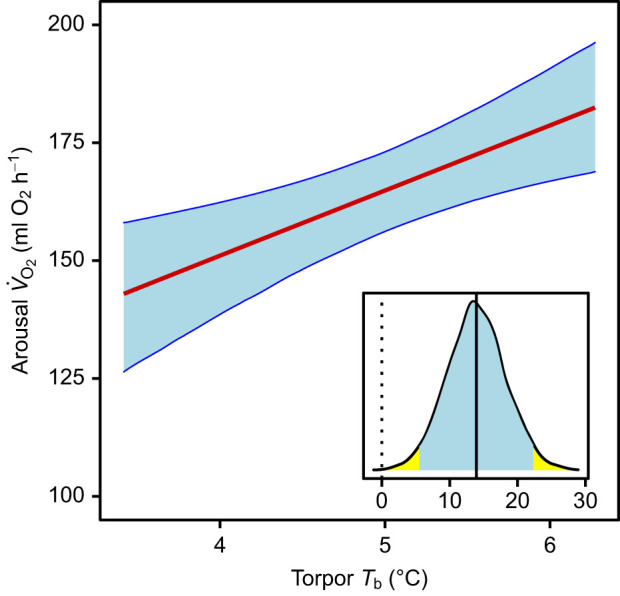
**Mean *V̇*_O_2__ during arousal as a function of mean *T*_b_ during the prior torpor bout.** Results of a Bayesian GLMM. Blue shaded areas indicate the 95% credible intervals of the predicted values. The inset shows the posterior distribution of the slope estimate, its mean (solid line) and its 95% credible interval (shaded); the dashed line indicates zero. Data from 94 torpor–arousal cycles in 22 animals.

Variation in arousal *V̇*_O_2__ was dominated by individual differences, as indicated by the conditional *R*^2^ (0.78) that was much greater than the marginal *R*^2^ (0.17). However, even slight fluctuations of temperature affected arousal *V̇*_O_2__. Interestingly, the average *T*_b_ during the previous torpor bout also had a positive effect on arousal *V̇*_O_2__ (Fig. 6, Table 1).

### *V̇*_O_2__ peaks during arousals

The mean maximum height of *V̇*_O_2__ during euthermia (*T*_b_>34°C) was 1.18-fold the mean *V̇*_O_2__ during the second half of arousal. Only in 30% of arousals was maximum *V̇*_O_2__ more than 20% greater than mean *V̇*_O_2__ during that period, i.e. constituted clearly visible peaks (e.g. Fig. 1).

## DISCUSSION

Our analysis shows that, during hibernation, torpor *V̇*_O_2__ was the single most important variable determining the duration of the torpid state. This is fully in line with the assumption that animals must arouse from torpor early, whenever an elevated metabolism facilitates a metabolic imbalance. Hence, these data, obtained from continuous measurements over entire torpor bouts, strongly support the idea of an underlying hourglass mechanism.

**Fig. 7.**
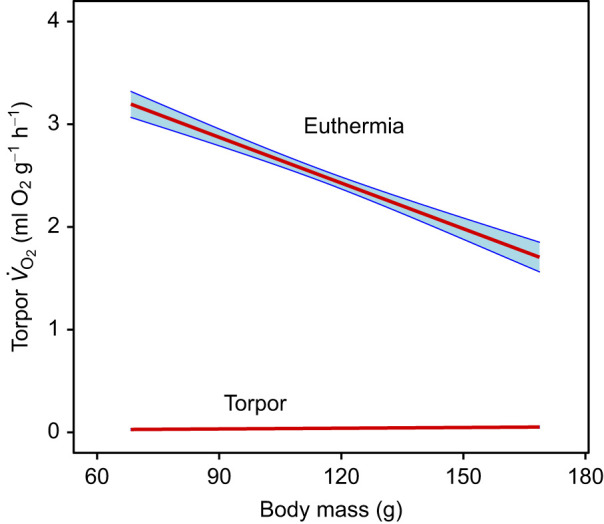
**Mass-specific *V̇*_O_2__ as a function of body mass in euthermic and torpid garden dormice.** Results of Bayesian GLMMs. Blue shaded areas indicate the 95% credible intervals of the predicted values. Data from 94 torpor–arousal cycles in 22 animals.

One argument against such an hourglass mechanism used in the past was the absence of body mass effects on torpor duration (Geiser and Ruf, 1995; Malan, 2010; Ruf and Geiser, 2015). The problem with all arguments involving body mass effects is, however, that minimum *V̇*_O_2__ during deep torpor is in fact virtually independent of body mass among hibernators. In comparative studies, depending on the dataset analysed, the slope of the regression of torpor *V̇*_O_2__ versus body mass is either indistinguishable from zero (Heldmaier et al., 2004) or minute, compared with the body mass dependency of basal metabolic rate in euthermic animals (Ruf and Geiser, 2015). In accordance with these comparative studies, the present data show that within a species, mass-specific *V̇*_O_2__ in torpor is indeed virtually independent of body mass (slope estimate: 0.0002±0.0001), while mean *V̇*_O_2__ strongly increased with smaller body mass in euthermic dormice (slope: −0.015±0.001; Fig. 7).

Thus, a simple hourglass mechanism is sufficient to explain torpor–arousal cycles, without the need to involve non-temperature-compensated cycles, which would be an unusual feature of a circadian rhythm (Rawson, 1960; Zimmerman et al., 1968). More importantly, there is increasing evidence for the central circadian pacemaker being arrested during hibernation (Hut et al., 2002; Ikeno et al., 2017; Revel et al., 2007; Williams et al., 2012). The problem with the alternative mechanism, the gradual development of some sort of metabolic imbalance, is that we know nothing about its nature. It seems likely, however, that the hourglass mechanism may involve protein turnover. Protein synthesis – with a few exceptions – is strongly depressed in hibernation (review in Storey, 2003), while protein degradation is low, but still ongoing (Van Breukelen and Martin, 2001; Yacoe, 1983). For example, it has been suggested that periodic arousals are due to the need to synthetize SERCA 2a, the calcium pump which is essential to maintain cardiac function in the torpid state (Ruf and Arnold, 2008). Alternatively, it has been proposed that periodic arousals are required for antibody production, i.e. to boost the immune system (Prendergast et al., 2002).

A common feature of these suggested targets is that the critical process is the loss of a substance during a torpor bout. We are not aware of any mechanism that would lead to the accumulation of a protein. At the same time, we know that protein synthesis completely ceases in deep torpor (Van Breukelen and Martin, 2001). Only at high body temperature during interbout euthermia are gene products restored (van Breukelen and Martin, 2002a; Van Breukelen and Martin, 2001). There are strong alterations over a torpor–arousal cycle, such as an almost complete depletion of circulating lymphocytes that is reversed rapidly upon arousal (Bouma et al., 2011). In fact, metabolomics have revealed a multitude of molecular changes, such as of amino acids, the metabolism of purine and pyrimidine, that of enzyme co-factors as well of various lipids, with observed metabolites reduced during torpor and increased upon arousal (Nelson et al., 2009).

Whatever the specific target, some insights can be gained for the relationships of torpor and arousal characteristics within a species. Notably, torpor bout duration in garden dormice was strongly dependent on torpor *V̇*_O_2__. Torpor *V̇*_O_2__ was in turn slightly correlated with torpor *T*_b_ (Fig. 4). Hence, it seems that *T*_b_ acts on bout duration via increasing torpor *V̇*_O_2__. This is in line with well-known effects of *T*_b_ on arousal frequency (e.g. Bieber and Ruf, 2009). Possibly, hibernators may use *T*_b_ as a proxy for metabolic rate, and for the speed by which a metabolic imbalance is approached. This is in agreement with measurements in ground squirrels, which led to the conclusion that *T*_b_ per se also contributes, along with *V̇*_O_2__, to determining the length of torpor bouts (Geiser and Kenagy, 1988). A role of *T*_b_ in controlling torpor bout duration was also indicated by experiments in arctic ground squirrels, in which a decline in *T*_a_ and *T*_b_ down to ∼0°C leads to increasingly longer maximum torpor bouts, while mean *V̇*_O_2__ showed only little variation (Buck and Barnes, 2000).

In the present experiments on garden dormice, torpor *V̇*_O_2__ was associated with *V̇*_O_2__ during the previous arousal (Table 1). It is likely that this correlation merely reflects individual differences in *V̇*_O_2__, both during torpor and arousal, which were strong (Table 1). *V̇*_O_2__ during arousals clearly increased with *T*_b_ in the previous torpor bout, and this was the strongest of all effects on arousal *V̇*_O_2__ (Table 1, Fig. 6). This finding suggests that any metabolic imbalance, e.g. metabolite depletion, that occurs faster at elevated *T*_b_ during torpor leads to increased *V̇*_O_2__ during the subsequent arousal.

There was a positive association between arousal duration and arousal *V̇*_O_2__ (Fig. 5A). In terms of the hourglass mechanism, this would mean that, within this species, increasing metabolic imbalances are not only corrected by merely increasing arousal duration but also by intensifying arousal *V̇*_O_2__. Partly, arousal duration was prolonged by additional peaks of *V̇*_O_2__ in the second half of the arousal (Fig. 5B). However, these peaks were not a prerequisite for torpor, as they were clearly identifiable only in 30% of all arousals.

There can be no doubt that the results of the present study, namely the dominant effect of metabolism on bout duration (Fig. 3), clearly indicate the *V̇*_O_2__-dependent development of an imbalance that is largely eliminated during arousal. The fact that both arousal and torpor bout duration, apart from some seasonal changes, are species-specific traits with limited variance (Ruf and Geiser, 2015) argues for an almost complete reset of this imbalance prior to the subsequent torpor bout. Only if the elimination of an imbalance during arousal is complete can we expect a more or less constant torpor bout duration (after its initial seasonal lengthening).

As outlined before, the missing allometric relationship of bout duration and *V̇*_O_2__ in torpor is only one of the reasons why an hourglass mechanism has not been universally accepted before. A simple further cause might be a lack of studies that gathered and analysed *V̇*_O_2__ and *T*_b_ throughout the entire torpor–arousal cycle, and not just by punctual measurements, possibly from different individuals. A third reason is that the relationship between bout duration and *V̇*_O_2__ is not always simple. Whenever hibernators maintain a large body-to-environment temperature gradient, i.e. thermoregulate in torpor, the resulting shortening of bout duration is smaller than expected from *V̇*_O_2__ at higher temperatures, when they keep minimal gradients (Buck and Barnes, 2000; Geiser and Kenagy, 1988). However, this may be readily explained if that fraction of metabolism allocated to pure heat production, in contrast to ‘basal’ torpor metabolic rate, does not contribute equally to the formation of an imbalance during torpor. A fourth reason is that torpor–arousal cycles, especially when they are relatively short but constant, when plotted like actograms, may resemble free-running circadian rhythms (e.g. Daan, 1973). As circadian rhythms are ubiquitous it seems natural to assume their involvement in the temporal control of hibernation too. However, it is now commonly accepted that torpor is an ancestral trait (Grigg et al., 2004; Kayser, 1961; Lovegrove, 2012a; Malan, 1996; Ruf and Geiser, 2015). There also seems to be a prevailing view that daily torpor is the ancient trait, whereas prolonged hibernation is considered an advanced, secondary adaptation (Grigg et al., 2004; Lovegrove, 2012b; Malan, 1996; Ruf and Geiser, 2015). In that case, the transition from daily to multiday torpor, often under conditions of constant darkness, i.e. hibernation, requires that any circadian rhythmic signal is supressed or that its generation is shut off. Apparently, this is exactly what happens at the onset of hibernation in autumn, and it is reversed in spring (Hut et al., 2002; Ikeno et al., 2017; Revel et al., 2007; Williams et al., 2012). This seems to be true for the central circadian pacemaker at least, but to the best of our knowledge hibernation is a centrally controlled phenomenon too (e.g. Florant and Heller, 1977; Ruby, 2003).

Alternatively, it has been argued that the simplest path to evolving a timer for hibernation cycles is via adaptation of an existing timing mechanism, the circadian system (van Breukelen and Martin, 2015). This led to the view mentioned above that a torpor–arousal cycle is considered a single non-temperature-compensated circadian day (Malan, 2010; van Breukelen and Martin, 2015). Indeed, the period of the circadian clock can be significantly modified by various metabolites, including mTOR signalling (Cao, 2018; Zhang et al., 2009). Circadian periods are lengthened by a few hours by these signals, typically to 27–28 h. However, in a free-living edible dormouse, for example, a circadian day would have to be lengthened from ∼24 h to ∼832 h on average during midwinter, as determined by temperature loggers (Hoelzl et al., 2015). Estimates for maximum torpor bout duration in certain bats are even above 2000 h (Ruf and Geiser, 2015). Thus, given these high natural torpor bout lengths and also the evidence that circadian rhythms are in fact temperature compensated well in hibernators and ectotherms (Rawson, 1960; Zimmerman et al., 1968), we consider this scenario rather unlikely.

Is an hourglass mechanism, that is, a rhythmic phenomenon caused by a process of accruing and subsequently decreasing physiological debts, unique among regulatory systems? Certainly not. A prime example is the sleep–wake cycle controlled by an increasing sleep deprivation during wakefulness, which is relieved during sleep. Whereas the current model of sleep regulation, which of course constitutes another hourglass mechanism, also involves circadian thresholds (Daan et al., 1984), such a circadian component is neither necessary nor desired in hibernation control of animals in constant darkness in underground burrows. Continued entrainment could be beneficial only in the rare hibernators that overwinter above ground.

Given the interspecific, highly significant, relationship between torpor *V̇*_O_2__ and bout duration (Ruf and Geiser, 2015), it seems that the hourglass mechanism determining the length of hibernation bouts is ubiquitous. Also, minimum torpor *V̇*_O_2__ is apparently subject to natural selection that decreases, while mean and maximum bout length increase in species living at higher latitudes under harsher conditions (Ruf and Geiser, 2015). The interspecific comparison also provides interesting insights into arousal duration. If arousal serves to correct a metabolic imbalance generated in torpor, e.g. to synthetize a crucial substance at high *T*_b_, this task should take longer if *V̇*_O_2__ during arousals is low. This is exactly what has been observed: the duration of interbout euthermia is sharply lengthened as body mass increases and basal metabolic rate declines. Interbout euthermia duration ranged from just 1.5 h in a 5 g bat to >28 h in a 3400 g alpine marmot (Ruf and Geiser, 2015).

### Conclusion

We conclude that control of torpor duration in hibernating garden dormice is governed by a progressively increasing metabolic imbalance that is eliminated during periodic arousals. Our data suggest that arousal from deep torpor may be activated once this imbalance, e.g. the depletion of a crucial metabolite, reaches a critical threshold. Comparative data suggest that this mechanism may be ubiquitous among hibernators. The threshold for arousal could be constant or under circadian fluctuation depending on the winter ecology of a species.

## References

[JEB243456C1] Bieber, C. and Ruf, T. (2009). Summer dormancy in edible dormice (*Glis glis*) without energetic constraints. *Naturwissenschaften* 96, 165-171. 10.1007/s00114-008-0471-z 19034404

[JEB243456C2] Bouma, H. R., Kroese, F. G. M., Kok, J. W., Talaei, F., Boerema, A. S., Herwig, A., Draghiciu, O., Buiten, A., Epema, A. H., van Dam, A. et al. (2011). Low body temperature governs the decline of circulating lymphocytes during hibernation through sphingosine-1-phosphate. *Proc. Natl. Acad. Sci. USA* 108, 2052-2057. 10.1073/pnas.1008823108 21245336 PMC3033260

[JEB243456C3] Buck, C. L., Barnes, B. M. (2000). Effects of ambient temperature on metabolic rate, respiratory quotient, and torpor in an arctic hibernator. *Am. J Physiol. Reg. Int. Comp. Physiol.* 279, R255-R262. 10.1152/ajpregu.2000.279.1.R255 10896889

[JEB243456C4] Bürkner, P.-C. (2017). brms: An R Package for Bayesian Multilevel Models using Stan. *J Stat. Softw.* 80, v080i01. https://www.jstatsoft.org/article/view/v080i01.

[JEB243456C5] Bürkner, P.-C. (2018). Advanced Bayesian multilevel modeling with the R Package brms. *R J* 10, 1. 10.32614.RJ-2018-017

[JEB243456C6] Cao, R. (2018). mTOR signaling, translational control, and the circadian clock. *Front. Genet.* 9, 367. 10.3389/fgene.2018.00367 30250482 PMC6139299

[JEB243456C7] Carey, H. V., Andrews, M. T. and Martin, S. L. (2003). Mammalian hibernation: cellular and molecular responses to depressed metabolism and low temperature. *Physiol. Rev.* 83, 1153-1181. 10.1152/physrev.00008.2003 14506303

[JEB243456C8] Daan, S. (1973). Periodicity of heterothermy in the garden doormouse, *Eliomys quercinus* (L.). *Neth. J Zool.* 23, 237-265. 10.1163/002829673X00067

[JEB243456C9] Daan, S., Beersma, D. G. M. and Borbély, A. A. (1984). Timing of human sleep: recovery process gated by a circadian pacemaker. *Am. J Physiol.* 246, R161-R178. 10.1152/ajpregu.1984.246.2.R161 6696142

[JEB243456C10] Daan, S., Barnes, B. M. and Strijkstra, A. M. (1991). Warming up for sleep?—ground squirrels sleep during arousals from hibernation. *Neurosci. Lett.* 128, 265-268. 10.1016/0304-3940(91)90276-Y 1945046

[JEB243456C11] Dausmann, K. H., Glos, J., Ganzhorn, J. U. and Heldmaier, G. (2004). Physiology: Hibernation in a tropical primate. *Nature* 429, 825-826. 10.1038/429825a 15215852

[JEB243456C12] Fernández-Verdejo, R., Ravussin, E., Speakman, J. R. and Galgani, J. E. (2019). Progress and challenges in analyzing rodent energy expenditure. *Nat. Methods* 16, 797-799. 10.1038/s41592-019-0513-9 31391589

[JEB243456C13] Fisher, K. C. (1964). On the mechanism of periodic arousal in the hibernating ground squirrel. In *Annales Academiae Scientiarum Fennicae Ser. A*, Vol. 71 (ed. P. Suomalainen), pp. 143-156. Helsinki: Suomalainen Tiedeakatemia.

[JEB243456C14] Florant, G. L. and Heller, H. C. (1977). CNS regulation of body temperature in euthermic and hibernating marmots (*Marmota flaviventris*). *Am. J Physiol. Reg. Int. Comp. Physiol.* 232, R203-R208. 10.1152/ajpregu.1977.232.5.R203 871176

[JEB243456C15] French, A. R. (1985). Allometries of the durations of torpid and euthermic intervals during mammalian hibernation: A test of the theory of metabolic control of the timing of changes in body temperature. *J Comp. Physiol. B* 156, 13-19. 10.1007/BF00692921 3836228

[JEB243456C16] Galster, W. and Morrison, P. (1976). Seasonal changes in body composition of arctic ground squirrel, *Citellus undulatus*. *Can. J Zool.* 54, 74-78. 10.1139/z76-008 1253012

[JEB243456C17] Geiser, F. and Kenagy, G. J. (1988). Torpor duration in relation to temperature and metabolism in hibernating ground squirrels. *Physiol. Zool.* 61, 442-449. 10.1086/physzool.61.5.30161266

[JEB243456C18] Geiser, F. and Ruf, T. (1995). Hibernation versus daily torpor in mammals and birds: physiological variables and classification of torpor patterns. *Physiol. Zool.* 68, 935-966. 10.1086/physzool.68.6.30163788

[JEB243456C19] Gelman, A., Carlin, J. B., Stern, H. S., Dunson, D. B., Vehtari, A. and Rubin, D. B. (2014). *Bayesian Data Analysis*. Boca Raton: CRC Press.

[JEB243456C20] Giroud, S., Stalder, G., Gerritsmann, H., Kübber-Heiss, A., Kwak, J., Arnold, W. and Ruf, T. (2018). Dietary lipids affect the onset of hibernation in the garden dormouse (*Eliomys quercinus*): implications for cardiac function. *Front. Physiol.* 9, 1235. 10.3389/fphys.2018.01235 30279661 PMC6153335

[JEB243456C21] Grigg, G. C., Beard, L. A. and Augee, M. L. (2004). The evolution of endothermy and its diversity in mammals and birds. *Physiol. Biochem. Zool.* 77, 982-997. 10.1086/425188 15674771

[JEB243456C22] Hall, M. (1832). On hybernation. *Phil. Trans. R. Soc. Lond.* 122, 335-360. 10.1098/rstl.1832.0017

[JEB243456C23] Heldmaier, G., Ortmann, S. and Elvert, R. (2004). Natural hypometabolism during hibernation and daily torpor in mammals. *Resp. Physiol. Neurobiol.* 141, 317-329. 10.1016/j.resp.2004.03.014 15288602

[JEB243456C24] Hoelzl, F., Bieber, C., Cornils, J. S., Gerritsmann, H., Stalder, G. L., Walzer, C. and Ruf, T. (2015). How to spend the summer? Free-living dormice (*Glis glis*) can hibernate for 11 months in non-reproductive years. *J Comp. Physiol. B* 185, 931-939. 10.1007/s00360-015-0929-1 26293446 PMC4628641

[JEB243456C25] Huber, N., Vetter, S., Stalder, G., Gerritsmann, H. and Giroud, S. (2021). Dynamic function and composition shift in circulating innate immune cells in hibernating garden dormice. *Front. Physiol.* 12, 620614. 10.3389/fphys.2021.620614 33746769 PMC7970003

[JEB243456C26] Hut, R. A., Barnes, B. M. and Daan, S. (2002). Body temperature patterns before, during, and after semi-natural hibernation in the European ground squirrel. *J Comp. Physiol. B* 172, 47-58. 10.1007/s003600100226 11824403

[JEB243456C27] Ikeno, T., Williams, C. T., Buck, C. L., Barnes, B. M. and Yan, L. (2017). Clock gene expression in the suprachiasmatic nucleus of hibernating arctic ground squirrels. *J Biol. Rhythms* 32, 246-256. 10.1177/0748730417702246 28452286

[JEB243456C28] Kayser, C. (1961). *The Physiology of Natural Hibernation*. Oxford: Pergamon Press.

[JEB243456C29] Kruschke, J. K. (2015). *Doing Bayesian Data Analysis: A Tutorial with R, JAGS, and Stan*. Academic Press.

[JEB243456C30] Larkin, J. E. and Heller, H. C. (1998). The disappearing slow wave activity of hibernators. *Sleep Res. Online* 1, 96-101. 11382864

[JEB243456C31] Lighton, J. R. B. (2008). *Measuring Metabolic Rates. A Manual for Scientists*. Oxford, New York: Oxford University Press.

[JEB243456C32] Lovegrove, B. G. (2012a). A single origin of heterothermy in mammals. In *Living in a Seasonal World. Thermoregulatory and Metabolic Adaptations* (ed. T. Ruf, C. Bieber, W. Arnold and E. Millesi), pp. 3-11. Berlin, Heidelberg, New York: Springer.

[JEB243456C33] Lovegrove, B. G. (2012b). The evolution of endothermy in Cenozoic mammals: a plesiomorphic-apomorphic continuum. *Biol. Rev.* 87, 128-162. 10.1111/j.1469-185X.2011.00188.x 21682837

[JEB243456C34] Lyman, C. P., Willis, J. S., Malan, A. and Wang, L. C. H. (1982). *Hibernation and Torpor in Mammals and Birds*. New York, San Diego: Academic Press.

[JEB243456C35] Malan, A. (1996). The origins of hibernation: a reappraisal. In *Adaptations to the Cold. Tenth International Hibernation Symposium* (ed. F. Geiser, A. J. Hulbert and S. C. Nicol), pp. 1-6. Armidale, New South Wales: University of New England Press.

[JEB243456C36] Malan, A. (2010). Is the torpor-arousal cycle of hibernation controlled by a non-temperature-compensated circadian clock? *J. Biol. Rhythms* 25, 166-175. 10.1177/0748730410368621 20484688

[JEB243456C37] Martin, S. L. and Epperson, L. E. (2008). A two-switch model for mammalian hibernation. In *Hypometabolism in Animals: Hibernation, Torpor and Cryobiology* (ed. B. G. Lovegrove and A. E. McKechnie), pp. 177-186. Pietermaritzburg, South Africa: University of KwaZulu-Natal.

[JEB243456C38] Nelson, C. J., Otis, J. P., Martin, S. L. and Carey, H. V. (2009). Analysis of the hibernation cycle using LC-MS-based metabolomics in ground squirrel liver. *Physiol. Genomics* 37, 43-51. 10.1152/physiolgenomics.90323.2008 19106184

[JEB243456C39] Prendergast, B. J., Freeman, D. A., Zucker, I. and Nelson, R. J. (2002). Periodic arousal from hibernation is necessary for initiation of immune responses in ground squirrels. *Am. J Physiol. Reg. Int. Comp. Physiol.* 282, R1054-R1082. 10.1152/ajpregu.00562.2001 11893609

[JEB243456C40] Rawson, K. S. (1960). Effects of tissue temperature on mammalian activity rhythms. *Cold Spring Harbor Symposia on Quantitative Biology* 25, 105-113. 10.1101/sqb.1960.025.01.010 13739935

[JEB243456C41] Revel, F. G., Herwig, A., Garidou, M. L., Dardente, H., Menet, J. S., Masson-Pévet, M., Simonneaux, V., Saboureau, M. and Pévet, P. (2007). The circadian clock stops ticking during deep hibernation in the European hamster. *Proc. Natl. Acad. Sci. USA* 104, 13816-13820. 10.1073.pnas.0704699104 17715068 10.1073/pnas.0704699104 PMC1959465

[JEB243456C42] Ruby, N. F. (2003). Hibernation: when good clocks go cold. *J Biol. Rhythms* 18, 275-286. 10.1177/0748730403254971 12932080

[JEB243456C43] Ruf, T. and Arnold, W. (2008). Effects of polyunsaturated fatty acids on hibernation and torpor: a review and hypothesis. *Am. J Physiol. Reg. Int. Comp. Physiol.* 294, R1044-R1052. 10.1152/ajpregu.00688.2007 18171691

[JEB243456C44] Ruf, T. and Geiser, F. (2015). Daily torpor and hibernation in birds and mammals. *Biol. Rev.* 90, 891-926. 10.1111/brv.12137 25123049 PMC4351926

[JEB243456C45] SAS Institute Inc. (2018). SAS/STAT User's guide, Version 15.1. SAS Institute Inc.

[JEB243456C46] Storey, K. B. (2003). Mammalian Hibernation. Transcriptional and translational controls. In *Hypoxia: Through the Lifecycle*, Vol. 543 (ed. R. C. Roach, P. D. Wagner and P. H. Hackett), pp. 21-38. New York: Kluwer/Plenum Academic. 14713112

[JEB243456C47] Strijkstra, A. M. (1999). Periodic euthermy during hibernation in the European ground squirrel: causes and consequences. *PhD thesis*, Rijksuniversiteit te Groningen.

[JEB243456C48] Strijkstra, A. M. and Daan, S. (1998). Dissimilarity of slow-wave activity enhancement by torpor and sleep deprivation in a hibernator. *Am. J Physiol. Reg. Int. Comp. Physiol.* 275, R1110-R1117. 10.1152/ajpregu.1998.275.4.R1110 9756541

[JEB243456C49] Tøien, Ø., Blake, J., Edgar, D. M., Grahn, D. A., Heller, H. C. and Barnes, B. M. (2011). Hibernation in Black Bears: Independence of Metabolic Suppression from Body Temperature. *Science* 331, 906-909. 10.1126/science.1199435 21330544

[JEB243456C50] Trachsel, L., Edgar, D. M. and Heller, H. C. (1991). Are ground-squirrels sleep-deprived during hibernation. *Am. J Physiol.* 260, R1123-R1129. 10.1152/ajpregu.1991.260.6.R1123 2058740

[JEB243456C51] Twente, J. W. and Twente, J. A. (1968). Progressive irritability of hibernating *Citellus lateralis*. *Comp. Biochem. Physiol.* 25, 467-474. 10.1016/0010-406X(68)90355-1 5653702

[JEB243456C52] Van Breukelen, F. and Martin, S. L. (2001). Translational initiation is uncoupled from elongation at 18°C during mammalian hibernation. *Am. J Physiol. Reg. Int. Comp. Physiol.* 281, 1374-1379. 10.1152/ajpregu.2001.281.5.R1374 11641105

[JEB243456C53] van Breukelen, F. and Martin, S. (2002a). Reversible depression of transcription during hibernation. *J Comp. Physiol. B* 172, 355-361. 10.1007/s00360-002-0256-1 12122451

[JEB243456C54] Van Breukelen, F. and Martin, S. L. (2002b). Invited review: molecular adaptations in mammalian hibernators: unique adaptations or generalized responses? *J Appl. Physiol* 92, 2640-2647. 10.1152/japplphysiol.01007.2001 12015384

[JEB243456C55] van Breukelen, F. and Martin, S. L. (2015). The hibernation continuum: physiological and molecular aspects of metabolic plasticity in mammals. *Physiology* 30, 273-281. 10.1152/physiol.00010.2015 26136541

[JEB243456C56] Wang, L. C. H. (1979). Time patterns and metabolic rates of natural torpor in the Richardson's ground squirrel. *Can. J Zool.* 57, 149-155. 10.1139/z79-012

[JEB243456C57] Williams, C. T., Barnes, B. M., Richter, M. and Buck, C. L. (2012). Hibernation and Circadian rhythms of body temperature in free-living arctic ground squirrels. *Physiol. Biochem. Zool.* 85, 397-404. 10.1086/666509 22705489

[JEB243456C58] Yacoe, M. E. (1983). Protein metabolism in the pectoralis muscle and liver of hibernating bats, *Eptesicus fuscus*. *J Comp. Physiol. B* 152, 137-144. 10.1007/BF00689738

[JEB243456C59] Zhang, E. E., Liu, A. C., Hirota, T., Miraglia, L. J., Welch, G., Pongsawakul, P. Y., Liu, X., Atwood, A., Huss, J. W., III, Janes, J. et al. (2009). A genome-wide RNAi screen for modifiers of the circadian clockin human cells. *Cell* 139, 199-210. 10.1016/j.cell.2009.08.031 19765810 PMC2777987

[JEB243456C60] Zimmerman, W. F., Pittendrigh, C. S. and Pavlidis, T. (1968). Temperature compensation of the circadian oscillation in *Drosophila pseudoobscura* and its entrainment by temperature cycles. *J Insect. Physiol.* 14, 669-684. 10.1016/0022-1910(68)90226-6 5655535

